# Comparing Satellite and Ground-Based Measurements of Environmental Suitability for Vector Mosquitoes in an Urban Landscape

**DOI:** 10.1093/jme/tjac145

**Published:** 2022-10-03

**Authors:** Andrea McMahon, Caio M B França, Michael C Wimberly

**Affiliations:** Department of Geography and Environmental Sustainability, University of Oklahoma, Norman OK, USA; Department of Biology, Southern Nazarene University, Bethany, OK, USA; Quetzal Education and Research Center, Southern Nazarene University, San Gerardo de Dota, Costa Rica; Department of Geography and Environmental Sustainability, University of Oklahoma, Norman OK, USA

**Keywords:** *Aedes albopictus* (Skuse) (Diptera: Culicidae), *Culex quinquefasciatus* (Say) (Diptera: Culicidae), remote sensing, urban habitat, environmental variables

## Abstract

Exposure to mosquito-borne diseases is influenced by landscape patterns and microclimates associated with land cover. These influences can be particularly strong in heterogeneous urban landscapes where human populations are concentrated. We investigated how land cover and climate influenced abundances of *Ae. albopictus* (Skuse) (Diptera: Culicidae) and *Cx. quinquefasciatus* (Say) (Diptera: Culicidae) in Norman, Oklahoma (United States). From June–October 2019 and May–October 2020 we sampled mosquitoes along an urban-rural gradient using CO_2_ baited BG Sentinel traps. Microclimate sensors at these sites measured temperature and humidity. We mapped environmental variables using satellite images from Landsat, Sentinel-2, and VIIRS, and the CHIRPS rainfall dataset. We also obtained meteorological data from the closest weather station. We compared statistical models of mosquito abundance based on microclimate, satellite, weather station, and land cover data. Mosquitoes were more abundant on trap days with higher temperature and relative humidity. Rainfall 2 wk prior to the trap day negatively affected mosquito abundances. Impervious surface cover was positively associated with *Cx. quinquefasciatus* and tree cover was negatively associated with *Ae. albopictus*. Among the data sources, models based on satellite variables and land cover data had the best fits for *Ae. albopictus* (*R*^2^ = 0.7) and *Cx. quinquefasciatus* (*R*^2^ = 0.51). Models based on weather station or microclimate data had weaker fits (*R*^2^ between 0.09 and 0.17) but were improved by adding land cover variables (*R*^2^ between 0.44 and 0.61). These results demonstrate the potential for using satellite remote sensing for mosquito habitat analyses in urban areas.

For effective control of disease-transmitting mosquitoes, it is essential to understand the distributions of vector mosquito species (Bousema et al. 2012, Hay et al. 2013, Pigott et al. 2015). Information about when and where mosquito abundance is highest can be used to target mosquito control efforts and disease prevention activities. Spatial and temporal variation of mosquito abundance is influenced by habitat availability and climate factors that affect their life cycles. Ecological models can help explain how different environmental variables influence species abundance and distribution. These environmental relationships can also be applied to predict species occurrence and abundance for locations where mosquito observations are not available (Guisan and Zimmermann 2000, Franklin 2010).

In central Oklahoma, the main medically important mosquitoes include species in the genus *Culex* as well as *Aedes albopictus* (Skuse) (Diptera: Culicidae). *Culex* mosquitoes are the primary vectors of West Nile virus in Oklahoma (Noden et al. 2015), where up to 176 human cases have been reported annually (Oklahoma State Department of Health 2021). *Culex quinquefasciatus* (Say) (Diptera: Culicidae), the species of the *Culex pipiens* complex that dominates in the southern United States, is a particularly important vector because it is prevalent in peri-domestic environments and likely to seek blood meals from humans (Reisen et al. 1990). *Aedes albopictus* is a competent vector for a variety of arboviruses, including yellow fever, dengue, chikungunya, Zika, and Eastern equine encephalitis. It has also been found to transmit West Nile virus (Holick et al. 2002, Sardelis et al. 2002, Zhang et al. 2022) and La Crosse virus (Gerhardt et al. 2001). *Aedes albopictus* is expanding its range into temperate areas (Farajollahi and Nelder 2009, Tjaden et al. 2021) and is likely to be responsible for future disease outbreaks (Manore et al. 2017) as it has already been in Europe (Vega-Rua et al. 2013). This species occurs at high densities in urban areas, putting them at risk of disease transmission (Manore et al. 2017).

Previous studies across different regions in Oklahoma have surveyed which mosquitoes are present and common in the area (Rozeboom 1942, Noden et al. 2015), assessed seasonal fluctuations in abundance (Bradt et al. 2019), and identified associations between land cover and mosquito abundance (Sanders et al. 2020). However, mosquito communities in Oklahoma are still understudied, and more research is needed to understand the patterns of vector mosquitoes and their associations with climate and land cover. The challenge of studying mosquito-environment relationships is compounded by the difficulty of measuring the diverse microhabitats that characterize heterogeneous urban landscapes (Pincebourde et al. 2016, Murdock et al. 2017, Wimberly et al. 2020). This fine-grained habitat variation can influence biologically relevant factors in the disease transmission cycle, such as vector density, survivorship, and biting rate (Hamer et al. 2011, LaDeau et al. 2015). Thus, studies based on environmental data from a single weather station or coarse-grained (>4 km) meteorological grids result in a scale mismatch between mosquito observations and environmental measurements. Field-based microclimate loggers can provide precise environmental data at specific locations. However, such studies include limited numbers of sample sites and may still not capture the full range of microhabitats.

Remote sensing offers an alternative source of environmental data. Earth-observing satellites provide measurements across large landscapes at consistent intervals and over long time periods. Satellite sensors have drastically improved over the past decades, and long-term archives of high-quality satellite imagery are expanding (Wulder et al. 2016). These developments, and the fact that various satellite data products are freely available, have led to the widespread use of satellite data to study spatiotemporal risk factors for a variety of vector-borne diseases (Dlamini et al. 2019, Malone et al. 2019, Wimberly et al. 2021). Satellite data products provide ecological variables that are relevant for mosquito ecology, including land cover classifications, vegetation indices, land surface temperature, and rainfall estimates. Many environmental remote sensing missions are global in scope, and thus satellite data can support research and applications in locations where *in situ* measurements are unavailable. However, satellite data have a range of spatial and temporal resolutions, and there can be a mismatch between the scales at which satellite data are retrieved and the scales of mosquito-environment interactions.

The goal of this study was to compare the relationships of mosquito abundance with different types of environmental measurements in an urban landscape. Our objectives were to: 1) identify environmental factors that influence abundances of the vector species *Cx. quinquefasciatus* and *Ae. albopictus,* and 2) evaluate how remotely sensed environmental data compares with *in-situ* data in relation to mosquito abundance. We achieved this goal by developing and comparing statistical models of mosquito abundance using environmental variables from microclimate loggers, local weather stations, and satellite Earth observations.

## Materials and Methods

### Study Area

The study area consisted of urban and rural areas within the city of Norman, OK. Norman has a population of 122,837 and a total area of approximately 460 km^2^ (U.S. Census Bureau 2019). The municipal boundary encompasses two Level III Ecoregions. The densely populated western part is within the Central Great Plains ecoregion, which is dominated by mixed-grass prairie and riparian woodlands. The less populated eastern part is within the Cross Timbers ecoregion, which consists of dense oak forests with open woodland areas (Woods et al. 2016). Norman, OK has a subtropical, semi-arid climate with average annual minimum temperature of 9.5°C, average annual maximum temperature of 21.8°C, and annual average precipitation of 987 mm (Oklahoma Climatological Survey 2021).

### Mosquito Trapping and Processing

**Fig. 1.**
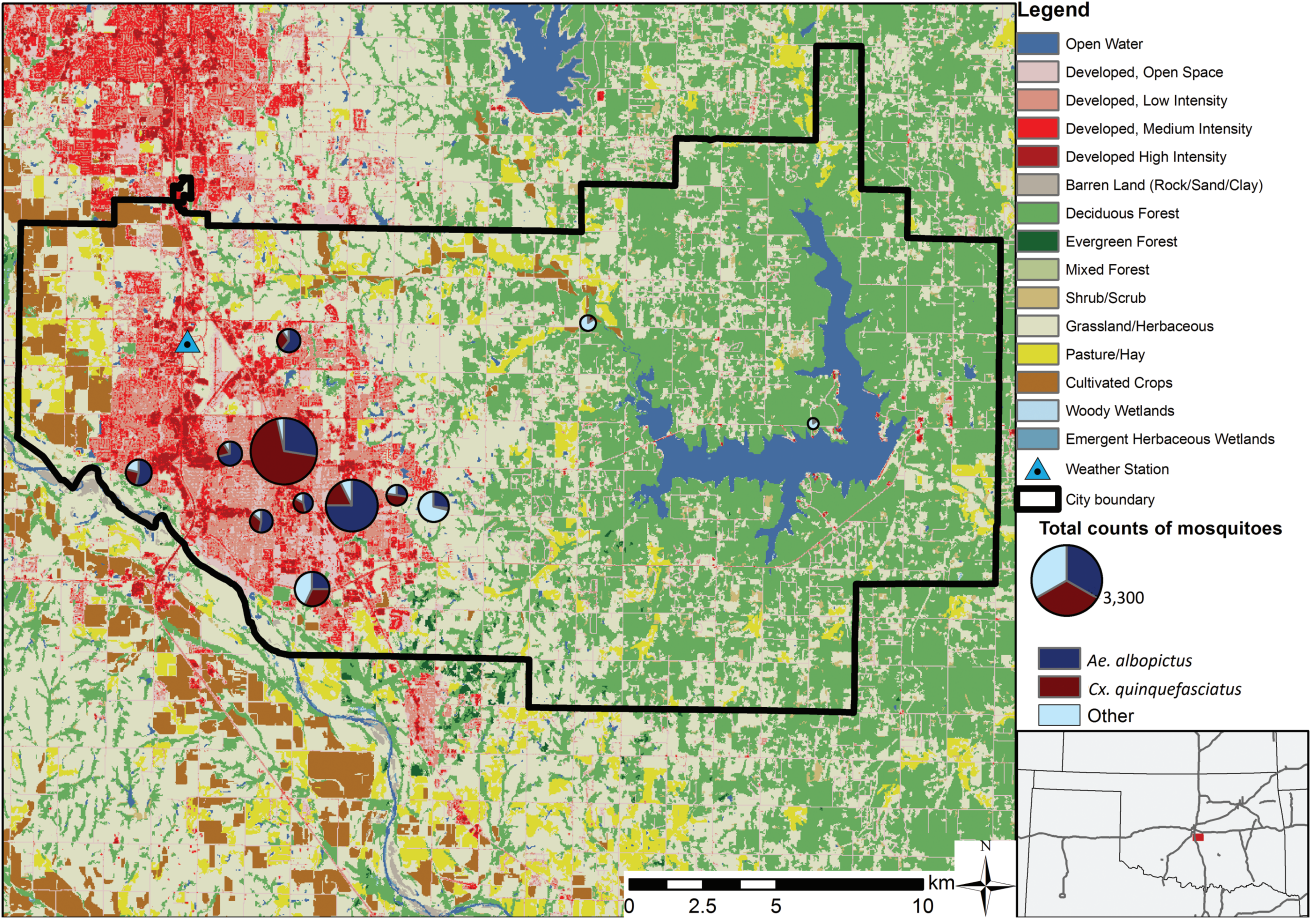
Land cover and locations of trap sites and the Oklahoma Mesonet weather station within the study area. The proportion of mosquito species collected at each trap site is shown as a pie chart and the size of each circle is proportional to the total mosquito abundance. Land cover data are derived from the 2016 NLCD dataset.

Mosquitoes were sampled between June and October in 2019 and May and October in 2020. We sampled at 11 consistent trap sites in both years, with a 12th site added in 2020, which resulted in a total of 255 trap nights. Trap locations were chosen to span a gradient from urban locations dominated by impervious surfaces to rural locations dominated by natural vegetation (Fig. 1). Sites were located in public parks, on residential property, and on the campus of the University of Oklahoma. Trapping was performed with BG Sentinel traps (Biogents AG, Regensburg, Germany). Because the octenol-based lure has been shown to underestimate mosquito species richness compared to CO_2_-baited traps (Wilke et al. 2019, Giordano et al. 2021), we added dry ice as a CO_2_ source to attract more mosquitoes. Mosquitoes were sampled bi-weekly, with traps deployed between 08:00 a.m. and 11:00 am and collected 24 h later. Adult female mosquitoes were examined under a dissecting microscope and morphologically identified using the taxonomic keys from Darsie and Ward (2005).

### Environmental Data Collection

We obtained static land cover data and dynamic data on relative humidity, precipitation, and temperature. Land cover data were retrieved from the National Land Cover Database (NLCD). Data sources for dynamic climate variables included microclimate loggers that were deployed in the field next to mosquito traps, satellite data from multiple sensors, and local weather station data from the Oklahoma Mesonet network (Table 1).

**Table 1.** Dynamic variables summarized for three data sources: microclimate data, satellite data, and weather station data

Dynamic variables	Units	Microclimate	Satellite	Weather Station
Tmin	C	daily minimum temperature	VIIRS nighttime land surface temperature	daily minimum temperature
Tmean	C	average of all temperature observations each day	NA	average of all temperature observations each day
Tmax	C	daily maximum temperature	VIIRS daytime land surface temperature	daily maximum temperature
RHmin	%	daily minimum relative humidity	NA	daily minimum relative humidity
RHmean	%	average of all relative humidity observations each day	NA	average of all relative humidity observations each day
RHmax	%	daily maximum relative humidity	NA	daily maximum relative humidity
Prec	mm	NA	CHIRPS precipitation	liquid precipitation measured each day
NDVI	index	NA	Normalized difference vegetation index from Sentinel 2 and Landsat OLI	NA
NDMI	index	NA	Normalized difference moisture index from Sentinel 2 and Landsat OLI	NA

#### Land Cover Data

We retrieved land cover data from the 2016 NLCD (Yang et al. 2003, 2018, Jin et al. 2019). In particular, we used the two NLCD products that measure imperviousness and canopy cover as a percentage of every 30-meter pixel. The two continuous land cover variables were summarized as focal means within a 1 km buffer around each trap site.

#### Microclimate Data

**Fig. 2.**
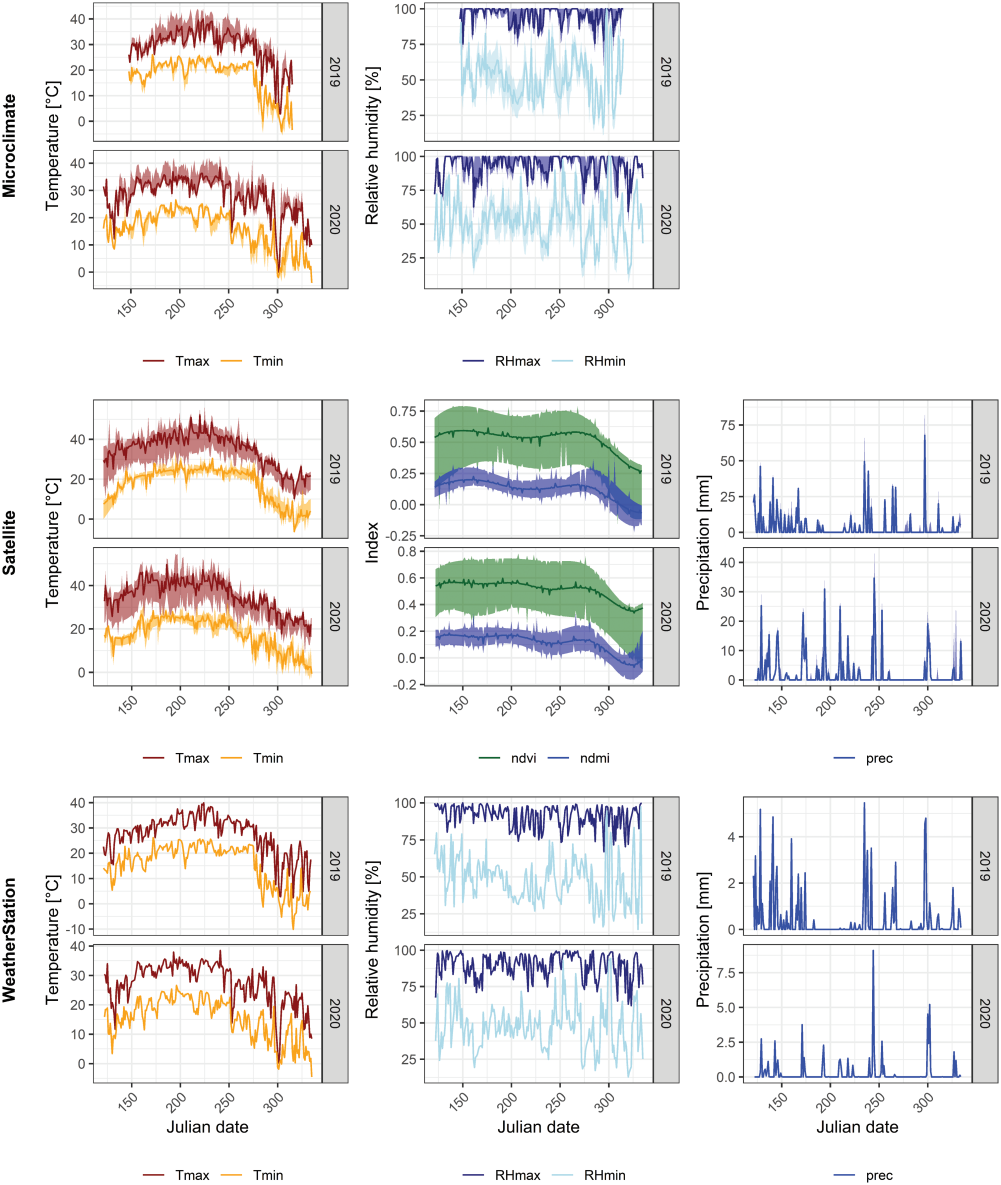
Environmental variables for temperature, spectral indices, precipitation, and relative humidity from microclimate loggers (top row), satellite remote sensing (middle row), and weather station data (bottom row). For microclimate data, the solid lines represent the daily median values and ribbons the minima and maxima across all sites. For satellite data, the graphs show daily interpolated values for a 1 km buffer zone around each trap with the solid lines representing the median values and the ribbons the minima and maxima across all sites. For weather station data, the solid lines show the daily values measured at the location of the weather station.

Microclimate loggers (RFID Track-it, Monarch Instruments) were deployed near trap sites to measure temperature and relative humidity. The loggers were equipped with radiation shields to reduce measurement errors (Tarara and Hoheisel 2007). We used two microclimate loggers at each residential trap site to measure conditions in the front and back yards. We deployed between 4 and 6 loggers within a radius of 100 m at each trap site on the university campus and in public parks. Loggers were placed at heights between 1 m and 1.5 m above ground and were located in areas with and without tree canopies to capture small-scale variability in microclimate. The loggers recorded data every 10 min from mid-May to mid-November in 2019 and 2020. Of the 41 loggers used in this study, ten malfunctioned and had to be replaced. In four instances, this led to missing data for the 4-wk period prior to the malfunction date. For each trap location, we calculated the daily minimum, mean, and maximum temperature and relative humidity. Values from all loggers around each trap site were interpolated via inverse distance weighting (Pebesma 2004). Daily values for all environmental data sources are displayed in Fig. 2.

#### Satellite Remote Sensing Data

We used datasets from several satellite sensors for comparison with weather station data and microclimate logger measurements. Daily rainfall measurements were derived from the Climate Hazards Group Infrared Precipitation with Stations (CHIRPS) dataset (Funk et al. 2015). This product provides daily disaggregated rainfall data at a spatial resolution of 0.05° × 0.05° (4.5 km at a Latitude of 35° N). Land surface temperature data were obtained from the Visible Infrared Imaging Radiometer Suite sensor (VIIRS) (Hulley and Hook 2018). We downloaded the VNP21A1D data product for daytime temperatures with observations at 01:30 p.m. local solar time and VNP21A2N for nighttime temperatures with observations at 01:30 a.m. local solar time. Land surface temperature data were available at daily intervals at a spatial resolution of 1km. We converted temperature values from Kelvin to Celsius. Data were masked via the products’ internal quality control layers to exclude cloud-contaminated pixels. Vegetation and moisture indices were calculated from harmonized Landsat and Sentinel-2 (HLS) data (Claverie et al. 2018). The dataset consists of Landsat 8, Sentinel 2A, and Sentinel 2B imagery, which are combined into a harmonized, analysis-ready surface reflectance dataset. Sentinel images are resampled to match the 30 m spatial resolution of Landsat 8 images. With observations from all satellites combined, the HLS dataset has a return interval of 2 to 3 d. From these HLS observations, we calculated a Normalized Difference Vegetation Index (NDVI) using the red and near-infrared reflectance bands (Tucker 1979), as well as the Normalized Difference Moisture Index (NDMI), using the near-infrared and the shortwave infrared reflectance bands (Gao 1996).

For each satellite variable, we computed focal means within a 1 km buffer around each trap site to have a standardized measure across all satellite products. This 1 km buffer reflects the maximum flight range of adult mosquitoes of the two target species, reported at approximately 700 m and 1 km for *Ae. albopictus* and *Cx. quinquefasciatus*, respectively (Verdonschot and Besse-Lototskaya 2014). For days with missing data, we used a linear regression model to impute those missing values for each of the satellite time series. We fit a linear model by robust regression using cyclical splines (Venables and Ripley 2002). We then replaced missing values with predicted values from the regression model.

#### Weather Station Data

We obtained meteorological data from the local weather station operated by the Oklahoma Mesonet network (Brock et al. 1995, McPherson et al. 2007). Variables included daily minimum, mean, and maximum values for temperature and relative humidity, as well as daily rainfall. The weather station is located at 35.2556° N, 97.4836° W in the northwestern part of Norman (Fig. 1). The distances between the weather station and the mosquito traps were between 2 and 20 km. Analyses based on weather station data assigned the same time series of meteorological variables to every trap site.

### Analysis

We analyzed mosquito-environment relationships by fitting statistical models for *Cx. quinquefasciatus* and *Ae. albopictus*. Many of the environmental variables from different data sources were not directly comparable to one another, and it was therefore not possible to specify the same model for all data sources. Instead, we used a variable selection procedure to identify subsets of variables and lags that had the strongest associations with mosquito abundance. To keep multivariate models parsimonious and comparable, we selected the four most important predictors for each of the three dynamic data sources (microclimate, weather station, satellite, and land cover). After identifying the most important variables, we carried out a multi-model comparison to assess fits of models based on data from different sources. Finally, we examined the effects of specific environmental variables in three final models that each included one of the three dynamic data sources (microclimate, weather station, or satellite data) combined with land cover.

All environmental variables were summarized for each trap day as well as over the 1, 2, 3, and 4 wk leading up to the trapping day. We then fitted generalized linear models using maximum likelihood estimation (Venables and Ripley 2002). To account for overdispersion, all models used a negative binomial distribution on untransformed count data (O’Hara and Kotze 2010). Variable selection was done with univariate models that were fitted for every combination of variable type (temperature, humidity, precipitation, and spectral index) and temporal summary (1 d, 1 wk, 2 wk, 3 wk, 4 wk). For each data source (microclimate, weather station, and satellite), we ranked these models by their Akaike information criterion (AIC) value. The AIC is a commonly used information criterion for model selection that penalizes overparameterization to pick the simplest of the best-fitting models (Bozdogan 1987, Akaike 1998).

We fitted multi-variable models for the dynamic data sources (microclimate, weather station, and satellite data) using the best four predictors from the univariate model comparisons as well as a separate model with only the two land cover variables. Three final models were fitted by adding the land cover variables to the microclimate, weather station, and satellite data models to account for purely spatial differences between trap sites. We generated time series graphs of the fitted values for these models to explore how the different data sources capture spatial and temporal variation in mosquito abundance. We calculated AIC statistics and Akaike weights (Wagenmakers and Farrell 2004) to compare the multivariate models for each mosquito species. The Nagelkerke pseudo-R^2^ (Nagelkerke 1991) was also calculated to allow comparison of models for different species. We generated the fitted values and computed the fit statistics using models that included only fixed effects, which allowed for a straightforward comparison of the environmental data sources. We also estimated the coefficients of the three final models using a mixed effects model that included a random effect for trap site to account for the clustered measurements at each location. To compare the effect sizes of the variables, we standardized them by rescaling to a mean of zero and a standard deviation of one.

## Results

We collected a total of 8,368 adult female mosquitoes across 11 trap sites in 2019 and 12 in 2020 (Table 2) with an average of 32.8 females per trap night. *Ae. albopictus* was the most abundant species over the entire time frame, with a total of 3,592 adult female mosquitoes and an average of 14.1 per trap night (42.9% of total counts). *Cx. quinquefasciatus* was the second most abundant species with a total of 3,256 adult female mosquitoes and an average of 12.7 per trap night (38.9% of total counts). *Psorophora ferox* made up 3.7% and *Ae. trivittatus* 2.72% of total abundance. All other species combined made up less than 2% of the total mosquito abundance. When broken down by year, *Ae. albopictus* was most abundant in 2019 with a total of 1,534 of females collected and an average of 15.5 per trap night (46.5% of 2019 counts). *Cx. quinquefasciatus* was most abundant in 2020 with a total of 2,438 females collected and an average of 15.6 per trap night (48.1% of 2020 counts).

**Table 2.** Mosquito abundances by species for 2019 and 2020. Displayed are abundances for all species, as well as their relative abundances

Species	2019	%	2020	%	total	%
*Aedes albopictus*	1,534	46.47	2058	40.62	3592	42.93
*Culex quinquefasciatus*	818	24.78	2438	48.12	3256	38.91
*Psorophora ferox*	224	6.79	85	1.68	309	3.69
*Aedes trivittatus*	183	5.54	45	0.89	228	2.72
*Culiseta inornata*	60	1.82	106	2.09	166	1.98
*Culex salinarius*	74	2.24	36	0.71	110	1.31
*Aedes vexans*	41	1.24	67	1.32	108	1.29
*Anopheles quadrimaculatus*	64	1.94	44	0.87	108	1.29
*Aedes triseriatus*	81	2.45	24	0.47	105	1.25
*Culex nigripalpus*	57	1.73	46	0.91	103	1.23
*Psorophora columbiae*	37	1.12	31	0.61	68	0.81
*Anopheles pseudopunctipennis*	25	0.76	12	0.24	37	0.44
*Culex tarsalis*	21	0.64	13	0.26	34	0.41
*Anopheles punctipennis*	7	0.21	13	0.26	20	0.24
*Psorophora cyanescens*	7	0.21	5	0.10	12	0.14
*Orthopodomyia signifera*	1	0.03	1	0.02	2	0.02
Culex spp	22	0.67	23	0.45	45	0.54
Aedes spp	20	0.61	2	0.04	22	0.26
Anopheles spp	1	0.03	1	0.02	2	0.02
Unknown	24	0.73	17	0.34	41	0.49
Total	3,301	100.00	5,067	100.00	8,368	100.00

**Fig. 3.**
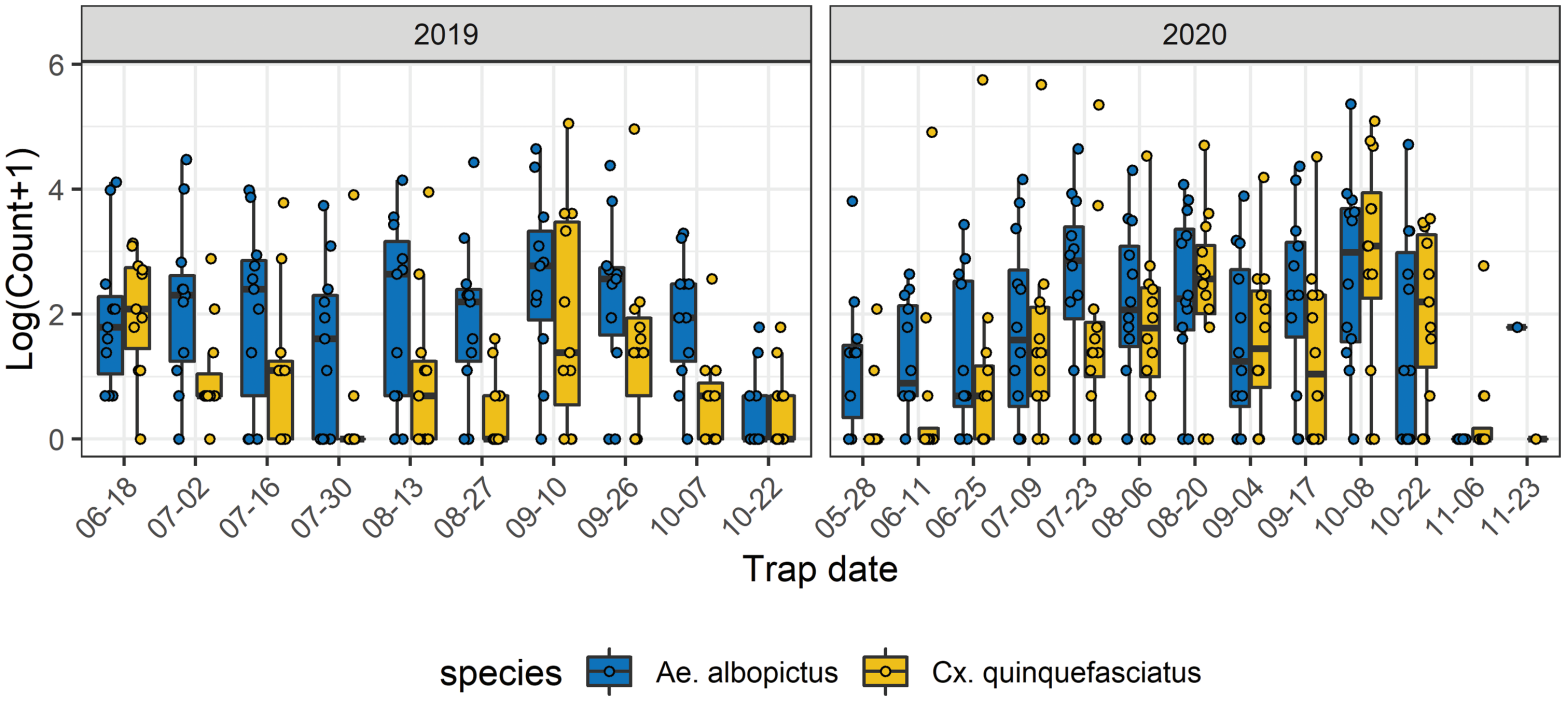
Mosquito counts per trap date for *Ae. albopictus* and *Cx. quinquefasciatus*. Box plots display the distribution of counts across trap sites for each trap date.

Species composition varied considerably by trap site and throughout the season (Fig. 3). *Ae. albopictus* had higher counts per trap week for almost the entire study period except for June 2019, August 2020, and October and November 2020, when we observed higher *Cx. quinquefasciatus* counts. Particularly in 2020, one trap site had consistently high counts of *Cx. quinquefasciatus*, that outnumbered *Ae. albopictus* even in the warmest summer months. Seasonal patterns were different in the 2 yr studied. In 2019, *Cx. quinquefasciatus* had the highest count in June, followed by lower numbers later in the summer and a spike in numbers in September. In 2020, *Cx. quinquefasciatus* numbers increased steadily between June and August, decreased in September, and increased again in October. *Ae. albopictus* counts had a comparable pattern in 2019, with highest numbers in September, and in 2020 with rising numbers between May and August and lower numbers in September followed by a late peak in early October.

When comparing models based on different data sources, the land cover models were the best fitting models with lowest AIC and highest pseudo R^2^ values for each species (Table 3). For *Ae. albopictus* the land cover model yielded an Akaike weight close to one and a pseudo R^2^ of 0.49. For *Cx. quinquefasciatus*, this model also yielded an Akaike weight close to one and a pseudo R^2^ of 0.41. Satellite time series data yielded the second-best model for both species with an Akaike weight of 9.69e-08 and a pseudo-R^2^ of 0.37 for *Ae. albopictus*, and an Akaike weight of 1.46e-11 and a pseudo-R^2^ of 0.22 for *Cx. quinquefasciatus*. For *Ae. albopictus*, the third best model was from Mesonet weather station data and the microclimate model had the weakest fit. For *Cx. quinquefasciatus*, the opposite was observed with microclimate data yielding a better fitting model than weather station data.

**Table 3.** Comparison of negative binomial fixed effect models for each data source individually

	Date source	AIC	delta AIC	Akaike weight	Pseudo R^2^	Model
*Aedes*	Land cover	1581	0	1.00E+00	0.49	canopy + impervious
	Satellite	1661	32.3	9.69E-08	0.37	ndmi_1wk + Tmax_4wk + Tmax_1dy + prec_2wk
	Weather station	1652	70.6	4.67E-16	0.17	Tmin_1dy + Tmax_2wk + RHmin_1dy + prec_2wk
	Microclimate	1662	80.8	2.85E-18	0.11	Tmin_1dy + Tmin_3wk + RHmin_1dy + RHmin_1wk
*Culex*	Land cover	1359	0	1.00E+00	0.41	canopy + impervious
	Satellite	1400	40.6	1.53E-09	0.22	ndmi_1wk + Tmax_4wk + Tmax_1dy + prec_2wk
	Microclimate	1409	49.9	1.46E-11	0.17	Tmax_1dy + RHmin_3wk + Tmax_2wk + RHmin_1dy
	Weather station	1421	62.5	2.68E-14	0.09	Tmax_1dy + prec_2wk + RHmax_2wk + Tmax_2wk

Adding land cover data on canopy cover and impervious surface cover to the models based on dynamic data sources led to lower AIC values and higher pseudo R^2^ values for all models (Table 4). Satellite time series data in combination with land cover data yielded an Akaike weight close to one and the highest R^2^ for both species. However, the difference between the best model and the second-best model was much larger for *Ae. albopictus* than for *Cx. quinquefasciatus*. The *Ae. albopictus* models had the overall highest pseudo-R^2^ values.

**Table 4.** Comparison of negative binomial fixed effect models for each data source in combination with land cover data

	Data source	AIC	delta AIC	Akaike weight	Pseudo R^2^	Model
*Aedes*	Satellite	1527	0	1.00E+00	0.7	ndmi_1wk + Tmax_4wk + Tmax_1dy + prec_2wk + canopy + impervious
	Weather station	1555	28.3	7.16E-07	0.61	Tmin_1dy + Tmax_2wk + RHmin_1dy + prec_2wk + canopy + impervious
	Microclimate	1571	43.8	3.08E-10	0.56	Tmin_1dy + Tmin_3wk + RHmin_1dy + RHmin_1wk + canopy + impervious
*Culex*	Satellite	1344	0	9.96E-01	0.51	ndmi_1wk + Tmax_4wk + Tmax_1dy + prec_2wk + canopy + impervious
	Microclimate	1356	11.2	3.68E-03	0.46	Tmax_1dy + RHmin_3wk + Tmax_2wk + RHmin_1dy canopy + impervious
	Weather station	1360	15.6	4.08E-04	0.44	Tmax_1dy + prec_2wk + RHmax_2wk + Tmax_2wk + canopy + impervious

**Fig. 4.**
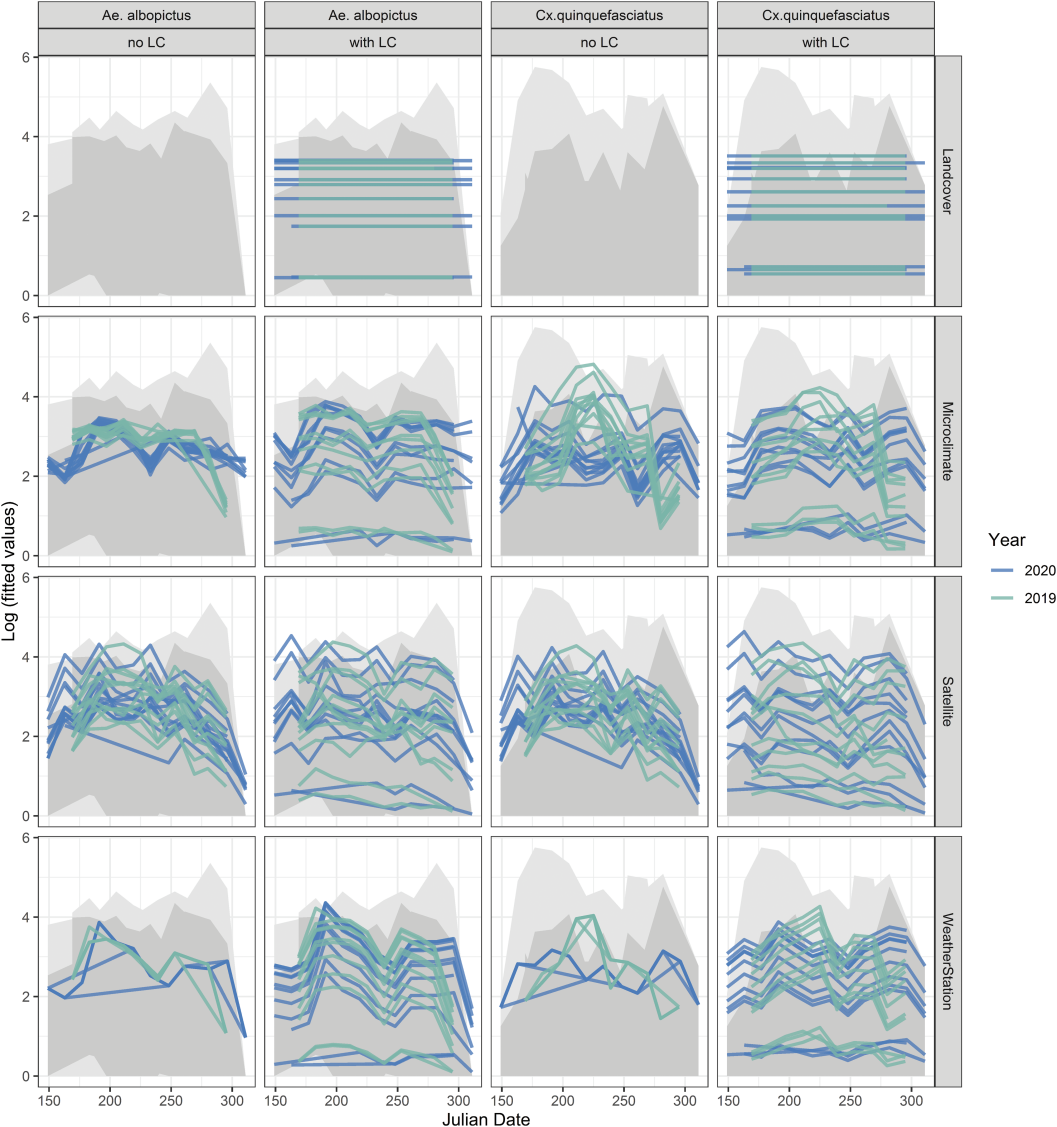
Fitted values from fixed-effects models based on land cover data, microclimate, satellite, and weather station data for *Ae. albopictus* and *Cx. quinquefasciatus*. Models based on microclimate, satellite, and weather station data were fitted with land cover data (with LC) and without land cover data (no LC). Different lines represent spatial variation among trap sites and years and each line represents variation within a season. Lines represent fitted values in 2019 and 2020. The grey shaded areas represent the range of mosquito counts across all traps for each year.

The fitted values from the multivariate models showed that land cover data captured spatial variability of both mosquito species, but not temporal variation (Fig. 4). Microclimate logger data captured almost no spatial variability of *Ae. albopictus* counts but did capture some spatial variability in *Cx. quinquefasciatus* counts. Mesonet variables, having been measured at a single location, captured temporal variability, but no spatial variability. Variables included in the satellite time series model were able to capture temporal, as well as spatial variability for both species. When land cover variables were added to the final models, all data sources were able to capture more spatial variability than comparable models without land cover variables.

**Fig. 5.**
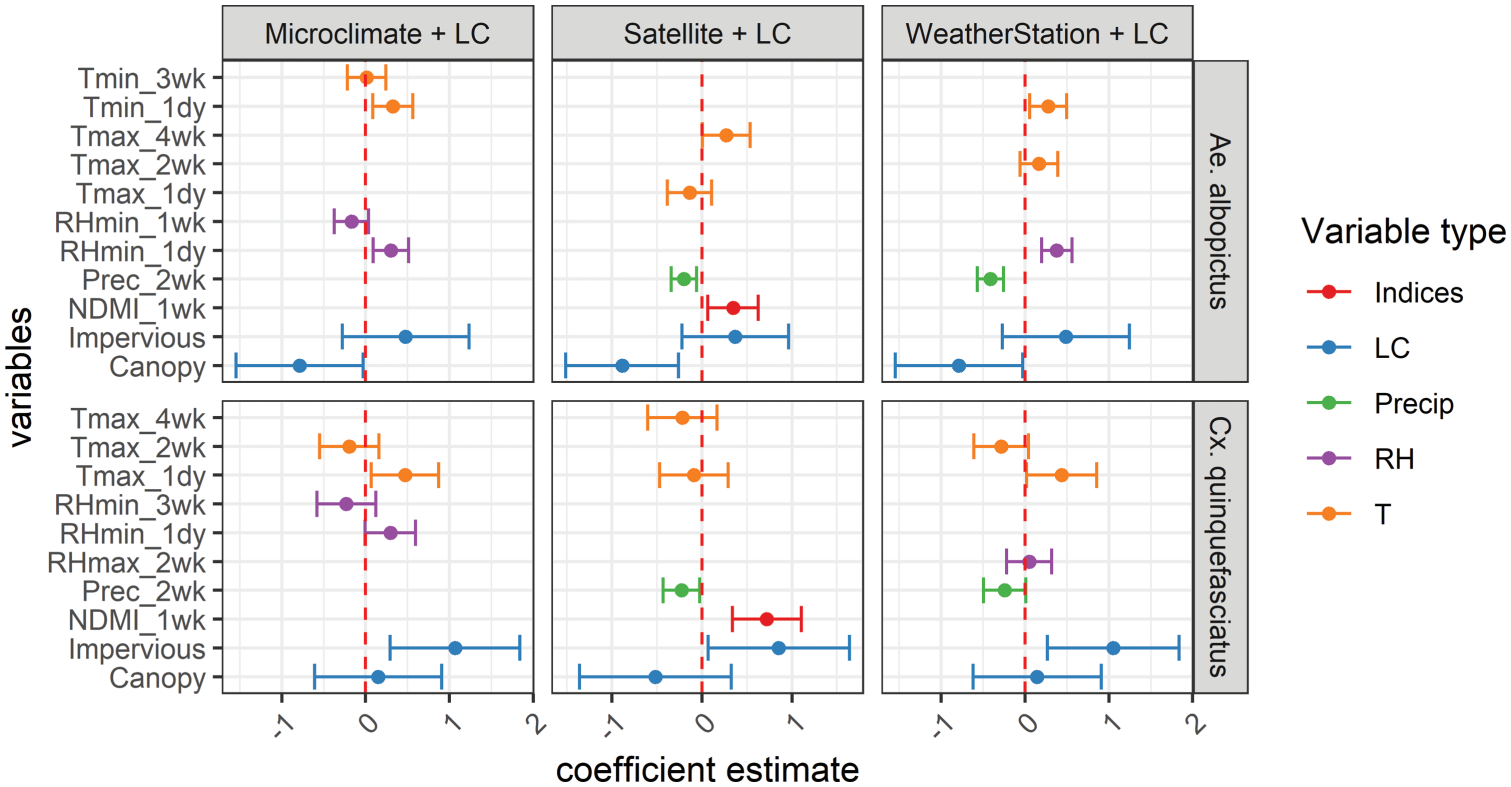
Coefficient estimates of mixed-effects models based on microclimate, satellite, and weather station data combined land cover data (LC) by species (*Ae.albopictus* and *Cx. quinquefasciatus*). Trap site was included as a random intercept. Variables are grouped by type (spectral indices (Indices), land cover (LC), precipitation (Precip), relative humidity (RH), and temperature(T)). Error bars indicate 95% confidence intervals.

In the models with microclimate and weather station data, temperature and relative humidity on the trap day had positive effects on mosquito abundances (Fig. 5). In the models based on satellite and weather station data, lower precipitation 2 wk preceding the trap date was associated with higher abundances of both mosquito species. In the models based on satellite data, higher vegetation moisture measured by harmonized Landsat-Sentinel 2 NDMI 1 wk before the trap date had positive effects on abundances of both mosquito species. Maximum (daytime) VIIRS land surface temperature 4 wk before the trap date was associated with higher abundances of *Aedes albopictus*. Impervious surface cover was positively associated with *Cx. quinquefasciatus* and tree cover was negatively associated with *Ae. albopictus*.

## Discussion

This study aimed to understand spatial and temporal variations in mosquito abundances for the two primary arboviral vector mosquitoes (*Ae. albopictus* and *Cx. quinquefasciatus*) in an urban area in central Oklahoma. Our models based on *in-situ* and satellite derived environmental variables and mosquito abundances for *Ae. albopictus* and *Cx. quinquefasciatus* showed that the two species have similar environmental associations in our study area. Both species had positive associations with temperature and relative humidity on the trap day. We attribute these relationships to influences of temperature (Veronesi et al. 2012, Nance et al. 2018) and humidity (Veronesi et al. 2012) on host-seeking behavior. The temperature variables that were identified by the variable selection procedure were all daily minimum and maximum temperatures rather than mean temperatures. This result is in accordance with previous findings that show how mosquito species react to ranges between minimum and maximum temperatures, as opposed to mean values (Carrington et al. 2013, Metelmann et al. 2019).

Both species had negative associations with precipitation at a 2-wk time lag. This time lag is possibly driven by the effect of breeding site flushing and ovipositional repellency from heavy rainfall events (Dieng et al. 2012). The flushing effect of breeding sites of anthropophilic urban mosquitoes was found to be stronger in cities with more build-up area, (Rydzanicz et al. 2016), as these tend to be areas where there is less infiltration and runoff is more extreme (Walsh et al. 2012). Particularly in central Oklahoma, where rainfall often occurs in extreme weather events, this effect may be exacerbated so that mosquito breeding is interrupted by flooding events, which may lead to decreased numbers of adult mosquitoes 2 wk after a heavy rainfall event. However, *Cx. quinquefasciatus* was positively associated with impervious surfaces. This effect has been previously observed for both, *Cx. quinquefasciatus* (Landau and van Leeuwen 2012), and for *Ae albopictus* (Shragai and Harrington 2019). We also found negative associations between *Ae. albopictus* and tree canopy cover, which reflect underlying associations with open areas dominated by mixtures of impervious surfaces and non-tree vegetation.

All four data types (land cover, microclimate, satellite, and weather station data) measured different variables, captured different amounts of spatial and temporal variability, and summarized the environment at different spatial scales. Static land cover variables measuring impervious surfaces and tree cover within a 1 km radius were the strongest predictors of abundance for both species and adding the land cover variables to models based on dynamic data (microclimate, satellite, or weather station data) substantially improved model fit. Of the dynamic data types, satellite data performed best, likely because spectral indices like NDVI (measuring greenness) and NDMI (measuring vegetation moisture content) capture aspects of land cover that have been shown to drive site-to-site variation and are additionally able to capture temporal variation. Both indices are positively correlated with canopy cover and negatively with impervious surfaces. However, even with spectral indices present in the satellite model, adding land cover variables still improved the model fit. Additionally, both satellite and weather station data provided information on precipitation, which revealed that precipitation 2 wk prior to the trap date negatively influenced mosquito abundance. The microclimate data, did not include precipitation, which may partially explain why the fits of the models based on microclimate data were weaker than those based on satellite data.

An important difference between the data types was the varying degree of spatial versus temporal information they provided, which affected their capacity to predict spatial and temporal variability in species abundance. Land cover data on canopy cover and impervious surfaces only captured spatial variability in species abundance, but these relationships were very strong for both species and models with land cover variables therefore had the best fits. This result highlights the importance of land cover variables as predictors of the geographic pattern of mosquito abundance. These results are in line with other studies showing the strong associations between different land covers and abundances of different mosquito species (Diuk-Wasser et al. 2006, Chuang et al. 2011, Landau and van Leeuwen 2012, Myer et al. 2020).

Satellite data captured spatial as well as temporal variation in species abundance for both species. This did not come as a surprise, as the satellite data include daily and near-daily time series of environmental variables for areas around trap sites (1 km radius) representing maximum movement distances. Models based on microclimate data captured similar temporal patterns as models based on weather station data. The microclimate models also captured some spatial variability for *Cx. quinquefasciatus*, but not for *Ae. albopictus*. This means that there was a spatial component of temperature and relative humidity that influenced *Cx. quinquefasciatus*, but not *Ae. albopictus*. *Cx. quinquefasciatus* was also related to impervious surfaces, which are known to affect microclimate conditions (Howe et al. 2017, Wimberly et al. 2020). Therefore, the spatial component explained by microclimate models likely reflects variation in microclimate that is associated with land cover. Weather station data by itself, measured at a single location, could not explain any of the spatial variability of abundance for either species. However, the dynamic weather station data can be combined with static land cover data to predict variation in mosquito abundance across both space and time (Chuang et al. 2011).

All data types were summarized at different spatial scales. The microclimate measurements were taken directly at the mosquito trap sites and were better predictors than weather station data for *Culex quinquefasciatus*, but not *Aedes albopictus*. However, the satellite data always had the best fit for both species even though they were summarized over a radius of 1km. Our results are in accordance with a recent study conducted in Florida, which found that the species distribution of *Ae. albopictus* was better predicted by coarser scale remotely sensed land cover variables than by finer scale microclimate data (Hopperstad et al. 2021). The weaker fits of the microclimate models compared to the satellite models could be explained by the fact that loggers placed in a small radius around mosquito traps may not be capturing the full range of microclimates within the flight range of the mosquitoes. Satellite data, on the other hand, provides a coarser-grained view on the landscape but were able to capture a larger extent that may better reflect the activity range of a mosquito. Considering that *Culex* species have been found to travel up to 3 km during their lifetime (Hamer et al. 2014), and *Ae. albopictus* up to 1 km (Vavassori et al. 2019), the scales at which medium resolution satellite missions like VIIRS and MODIS gather data (250–1000 m), appear to be suitable for predicting mosquito abundance.

The results of this research and similar studies of mosquito abundance are contingent upon the sampling methods, including the geographic extent, site density, time period, and frequency. Species composition and abundance of captured mosquitoes also depends on the trap and the bait used. BG Sentinel traps have a lure that was designed for artificial container breeding *Aedes* species and are known to underestimate other mosquito species. We added dry ice from CO_2_ as additional bait, which has been shown to equally attract *Ae. aegypti* and *Cx. quinquefasciatus* and increase the overall abundance and diversity of mosquitoes caught (Wilke et al. 2019). However, we acknowledge that the different trapping methods, such as CDC light traps or gravid traps, would likely result in different species distributions. Furthermore, our analysis was conducted over two summer seasons with bi-weekly sampling. Even though we captured considerable temporal variability in mosquito abundance and environmental conditions, future research over longer time periods and more frequent sampling would be desirable to confirm our results and to further investigate variation within and between years. Similarly, the 12 trap sites captured a gradient from highly urbanized sites, rural areas and uninhabited parklands, but a larger number of traps over a wider range of microhabitats could offer further insights. The microclimate measurements were taken using small, inexpensive data loggers that have an accuracy of up to ± 0.5°C (Wimberly et al. 2020). Some microclimate loggers failed and had to be replaced mid-season, which resulted in missing data and increased the uncertainty in our microclimate measurements.

This study examined measurements of the physical environment that influenced habitat suitability for mosquitoes. In addition, it is known that socioeconomic factors such as the age of a neighborhood (Spence Beaulieu et al. 2019), household income and education level (Little et al. 2017, Whiteman et al. 2019), as well as ownership and use status (Lambin et al. 2010) can influence persistence and quality of larval habitats and mosquito species distributions within heterogeneous urban areas. For example, the abundance of uncovered trash containers (e.g., plastic cups, Styrofoam bowls, plastic bags, cans) and old car tires is higher in low-income areas, whereas containers for recreational use (e.g., small children’s pools, sandboxes, sporting equipment and toys) are the main breeding sites in high income areas (LaDeau et al. 2013). Differences in water accumulation in rain-fed unmanaged containers and containers that are purposefully watered can also lead to seasonal variation in mosquito abundance between neighborhoods with different socio-economic status (Becker et al. 2014). Socioeconomic factors may also influence how likely it is that households or neighborhood associations control for mosquito populations. Although these factors were outside the scope of our study, future studies could include additional data on irrigation, mosquito control practices, and artificial containers at household and neighborhood levels.

In conclusion, impervious surfaces and tree cover have strong influences on the abundances of *Ae. albopictus* and *Cx. quinquefasciatus* in Norman, Oklahoma. Our results also indicate that satellite-based measurements of land surface temperature, rainfall, and vegetation produced the best fitting models of mosquito abundance and were able to capture spatial as well as temporal variation. The fits of models based on satellite remote sensing, microclimate and weather station data all improved substantially when they were combined with classified land cover maps derived from satellite data. In parts of the world where ground-based environmental monitoring data are sparse, satellite remote sensing provides essential data for predicting mosquito abundance across space and time. Even where *in-situ* measurements are available, satellite observations provide unique, spatially explicit environmental measurements that can have stronger relationships with mosquito abundance than data from ground-based weather stations and microclimate data loggers.
